# Across-Time Change and Variation in Cultural Tightness-Looseness

**DOI:** 10.1371/journal.pone.0145213

**Published:** 2015-12-18

**Authors:** Anne Mandel, Anu Realo

**Affiliations:** 1 Brain Research Unit, O.V. Lounasmaa Laboratory, Aalto University, Helsinki, Finland; 2 Department of Psychology, University of Tartu, Tartu, Estonia; Mälardalen University, SWEDEN

## Abstract

Cultural tightness-looseness, a dimension which describes the strength, multitude, and clarity of social norms in a culture, has proved significant in explaining differences between cultures. Although several studies have compared different cultures on this domain, this study is the first that targets both within-country differences and across-time variation in tightness-looseness. Using data from two nationally representative samples of Estonians, we found that the general tightness level had changed over a period of 10 years but the effect size of the change was small. A significant within country variance in 2002 had disappeared by 2012. Our results suggest that tightness-looseness, similarly to cultural value orientations, is a relatively stable and robust characteristic of culture–that is, change indeed takes place, but slowly. Future studies about across-time change and within-country variance in tightness-looseness should target more culturally diverse and socially divided societies.

## Across-Time Change and Variation in Cultural Tightness-Looseness

Culture is often seen as a set of learned and shared meanings that distinguishes one group of people from another [1, 2]. Since the emergence of comparative studies of culture, social scientists have been trying to find general cultural dimensions or orientations, not just colorful single details, to capture major cross-cultural differences (e.g., [3–10]). In this paper, we focus on tightness-looseness as a promising dimension for understanding cultural variation [11].

Tight cultures have clear and pervasive social norms and severe sanctions for deviant behavior, whereas loose cultures have weak norms and high tolerance of deviant behavior. A cross-cultural study involving 33 countries [12] demonstrated that both ecological and historical factors, as well as proximal and contemporaneous processes, vary across tight vs. loose nations. Tighter nations, for instance, have encountered more historical or ecological threats, like wars, natural disasters, and famine. Cultural tightness has also been related to less open media, higher levels of religiosity, stronger laws and regulations, stricter punishments, and lower crime rates [12], as well as to lower levels of CEO discretion [13], and even higher stock market comovement [14]. Relative to moderate nations, both very tight and very loose nations have lower happiness, worse health outcomes, and gross domestic product per capita [15]. A recent study [16] showed that there is a wide variation in tightness–looseness, not only across nations, but also across the 50 states of the United States, which is also associated with different ecological and historical factors, psychological traits, and state-level outcomes. On an individual level, higher tightness has been related to higher constraints in everyday situations [17], higher cautiousness, and greater need for self-regulation and structure [12].

However, despite a large number of studies on tightness-looseness over the past decade, several important questions remain unanswered. First, the stability of tightness-looseness scores across time has so far not been assessed. This is, however, an important theoretical question which needs to be answered in order to fully understand how the level of tightness-looseness in societies is changed or maintained. Secondly, as far as we know, comparative studies of tightness-looseness have been rarely conducted using large representative samples. Instead, relatively small convenience samples have been used (e.g., [12, 18, 19]), thus casting doubt over the generalizability of findings to a wider population [20]. (There is a recent study on cultural tightness-looseness that was carried out in representative national samples of 68 countries but in this study, authors did not directly measure tightness-looseness but proposed new indices of cultural tightness-looseness that were based on the variation (i.e., standard deviation) in different values and behavioral practices within a country [21].) Finally, relatively small and possibly biased samples have also prevented comparison of different subgroups (i.e., men vs. women, more educated vs. less educated, younger vs. older, etc.) within cultures on their level of tightness-looseness.

In the current paper, we advance the field by studying changes in tightness-looseness in Estonia over 10 years in two large nationally representative samples. This enables us to (a) study across-time changes in tightness-looseness; (b) draw conclusions about the whole population of a country; and (c) compare differences in tightness-looseness across different social subgroups (by gender, age, education, place of residence, and language used to complete the questionnaire).

### The importance of studying across-time variation in tightness-looseness

Cultures change over time, with gradual yet fundamental changes occurring in people’s basic values–which are often seen as the quintessence of culture–in response to socioeconomic modernization and democratization, as well as contact with other cultures [22, 23]. Longitudinal studies have even shown that cultural change in values, at least to a certain extent, can be predicted on the basis of a society’s socioeconomic development and religious heritage [24]. Rising socioeconomic status, in particular, tends to enforce secularization [22], as well lead to higher levels of individualism [25]. There are also examples of short-lived, but dramatic, events, like 9/11 in the United States (US), eliciting changes in the social environment and people’s behavior [26, 27]. These results raise the question of how much other characteristics of a culture, such as tightness-looseness, change over time.

### From a cross-cultural perspective to an examination of within-country variation

Cross-cultural psychologists often treat countries as homogeneous units, implicitly assuming that every member of a certain culture shares the same set of cultural characteristics with other members of that culture [28]. Although, in many cases, such a generalization may be justified, there is still evidence that intracultural differences are often larger than variations between cultures (e.g., [29, 30]). Indeed, a broad analysis of various behavioral measures [31] showed that samples consisting of young western college students, for instance, are remarkably different from other social subgroups in the same country, even in simple visual perception tasks, but also in moral reasoning, self-concept, and heritability of IQ (these being just a few examples). Furthermore, although the US is regarded as a prototypical individualistic culture, studies have shown that the Southern US is rather collectivistic, as opposed to the more individualistic Great Plains and Mountain West areas [32]. Similarly, a recent study [16] showed that there is large variation in tightness–looseness at the state level in the US, with the Southern region being the tightest and the Northeast and the Western regions the loosest.

### Aims of the present study

The aims of the current study are a) to examine change in tightness-looseness scores over time and b) to study within-country variance in tightness-looseness in Estonia, by using data from one large-scale nationally representative survey of Estonian residents conducted in 2002 (the Estonian Survey of Culture and Personality, ESCP2002; *N* = 1,582) and one in 2012 (the 6th wave of the European Social Survey, ESS2012; *N* = 1,883).

The example of Estonia (1.3 million inhabitants, population density 29/km^2^) is particularly interesting, because it was the second loosest country (after the Ukraine) in a 33-country comparative study of cultural tightness-looseness [12]. It has been argued [21] that norms, values, and behaviors should show greater variability in loose cultures than in tight cultures, which are more homogeneous and do not allow for much variation in values and acceptable behaviors. A study showed, for instance, that different population groups (inhabitants of an isolated island, army conscripts, housewives with many children, etc.) in Estonia have remarkably different patterns of collectivism [33]. Earlier research also found considerable differences in both value preferences (e.g., [34]) and the strength of collectivistic attitudes [35] between the Estonian- and Russian-speaking populations in Estonia. Such results clearly illustrate the importance of using samples that are representative of the whole population and paying attention to within-country differences, both at the level of geographic regions and among different subgroups in society.

Considering the social, political, and economic changes that have taken place in Estonia during the past 25 years, Estonia also makes an interesting example for examining the stability of tightness-looseness over time. In early 2002, when our first set of data was collected, Estonia was still going through major changes in the structure and organization of society, having regained independence in 1991. After going through the stages of radical reform (1992–1995) and economic stabilization as well as technological modernization (1995–1999), the years 1999–2004 in Estonia were mainly characterized by integration with Europe [36]. Joining the EU and NATO in 2004 can be viewed as the end of the transition period in Estonia. During the years 2005–2007, the country enjoyed around 10% growth in GDP per year and this period was defined by an increase in economic wellbeing as well as in general life satisfaction [37]. In 2007, however, the economy began to cool down. While, during the transition phase, striving for personal autonomy and little concern for the “common good” were prevalent attitudes in Estonia [38], the economic crisis shifted attention to social development and intensified calls for social reforms [39]. It can be hypothesized that the financial restrictions applied during the economic crisis as well as the new rules and regulations that came into effect after joining the EU and NATO have made the general environment in which people act less flexible (those being only a few examples of the processes that took place in the relatively young democracy). The same changes can also be viewed in the opposite way: as diminishing the borders between countries and encouraging more intercultural interaction and thereby blurring existing national social norms. In terms of cultural values, the importance of hedonism, material well-being, self-direction, and close relations steadily grew in Estonia from 1985 to 2008 [40, 41]. However, the major shift in values took place right after re-independence in 1990s, and the period of 2000s could rather be described in terms of relative value stability [42, 43].

## Method

### Participants

#### ESCP2002

The first sample came from a study of social capital, cultural value dimensions, and identity in Estonia in 2002 (Estonian Survey of Culture and Personality, ESCP2002). The sample was randomly selected from the National Census and was representative of the Estonian population in terms age, gender, ethnicity, place of residence, and educational level (for a sample description, see also [44, 45]). Altogether, 1,753 respondents aged 15–74 participated in ESCP2002. Complete data (with no missing values) were available for 1,582 people (889 female, 693 male; mean age 43.5, *SD* = 17.4); 1,328 (84%) of the respondents filled in the questionnaire in Estonian and 254 (16%) in Russian. Nineteen per cent of participants had completed primary (1–9 years), 45% secondary (10–12 years), and 36% tertiary (13 years or more) education. Twenty-six per cent of respondents lived in a big city or its suburbs, 30% in a small city or town, and 44% on a farm or in a village.

#### ESS2012

The second sample is part of the European Social Survey round 6 (ESS2012), carried out in 30 European countries (http://www.europeansocialsurvey.org). The sample was again randomly selected from the Estonian population and was representative of all residents of private households, aged 15 and over, regardless of their citizenship, ethnicity, or language. In total, 2,095 respondents aged 15–94 years participated in the ESS 6th round survey in Estonia. Data with no missing values were available for 1,883 participants aged 15–74 years (1,063 female, 820 male; mean age 44.9, *SD* = 16.7 years). Seventy per cent (*n* = 1,324) of the respondents answered in Estonian and 30 per cent (*n* = 559) in Russian. Eleven per cent of the participants had at least primary, 38% secondary, and 51% tertiary education. Thirty-six per cent of respondents lived in a big city or its suburbs, 33% in a small city or town, and 31% on a farm or in a village.

It was not necessary to seek approval for the present study from the Ethics Review Committee on Human Research of the University of Tartu because we were using secondary data from existing social surveys in our analyses. Both datasets (ESCP2002 and ESS2012) have been carefully cleaned of all identifying information to ensure anonymity of the data files and therefore, it is impossible that the data could be linked back to the individuals from whom it was originally collected. In both surveys (i.e., ESCP2002 and ESS2012), verbal informed content was obtained and participants were assured of the confidentiality, privacy, and anonymity of any information shared.

### Measures

#### Tightness-Looseness Scale (TLS)

Both in the ESCP2002 and the ESS2012, participants were asked to complete a 6-item Tightness-Looseness Scale (TLS; [12]). The scale targets the strength, clarity, and number of social norms, the degree of tolerance for deviance from these norms, and overall compliance with social norms in a country, with items like “There are many social norms that people are supposed to abide in this country” and “In this country, if someone acts in an inappropriate way, others will strongly disapprove” (the items of the TLS are shown in S1 Appendix). The instructions given to respondents were that the statements refer to the country as a whole and that the statements refer to “social norms,” which are standards for behavior that are generally unwritten. The respondents rated the items on a 6-point Likert-type scale, ranging from “Strongly disagree” (1) to “Strongly agree” (6). The scale was translated into Estonian by the second author (accuracy verified by back-translation to the original language) and to Russian by a bilingual expert of both the Estonian and Russian languages (see S1 Appendix).

In a 33-nation study of tightness-looseness [12], the TLS showed a single-factor structure, with the first factor explaining 62% of underlying variance in an exploratory factor analysis (including data from all 33 nations, *N* = 6,823). Factor loadings were .68 or greater, with the exception of (reverse-coded) item #4, which had a loading of .26, in the expected direction. The national-level reliability of the scale in the abovementioned study [12] was very good (Cronbach’s α = .85), and validity across nations was also demonstrated by showing that the factor structure obtained across nations is equivalent to the factor structure found separately in each nation.

The following variables, which were available in both datasets, were selected for the defining subgroups: (a) the language in which the respondent completed the questionnaire (Estonian or Russian); (b) gender; (c) age; (d) education level; and (e) place of residence (from big city to village or farm). These particular variables were chosen because previous research about social norms, values, and personality has indicated these as potential sources of intracultural diversity (e.g., [22, 34, 46–50]).

### Preliminary data analysis

#### Internal consistency

Internal consistency of the TLS was assessed by Cronbach’s α. Differently from an earlier study [12], item #4 (“People in this country have a great deal of freedom in deciding how they want to behave in most situations”) did not work as expected in the current study: although it was designed to be a reverse-coded item, it showed positive correlations with the other five items in the scale (see S1 Table). Item #4 also had, on average, the lowest correlations with the other items in the scale. To test whether the problems with item #4 might reflect difficulties in sentence comprehension, we checked whether item #4 would fit the expected model among highly educated respondents. Among people who had a higher education, the average correlation between reversed item #4 and the other TLS items was -.04 in the ESCP2002 and -.22 in the ESS2012 data. Average correlations between other TLS items varied between .19 and .34 among higher educated respondents in the ESCP2002 sample and between .29 and .40 in the ESS2012 sample, showing that the item did not work as expected, even among highly educated respondents.

Cronbach’s α for the 6-item scale (item #4 reversed, according to the expected model) was .47 in the ESCP2002 data and .51 in the ESS2012 data. When item #4 was left out, Cronbach’s α increased to .61 for the ESCP2002 data and to .71 for the ESS2012 data. Therefore, item #4 was left out in all further analyses. Excluding any other single item did not improve internal consistency of the scale. Thus, we calculated the mean tightness score across five items of the TLS (higher scores indicate greater tightness).

#### Model fit

A confirmatory factor analysis (CFA; Maximum likelihood method) was used to verify whether the single-factor tightness-looseness model [12] fit the current data. In both datasets, the 5-item single-factor model reached an acceptable fit [51], i.e., AGFI and CFI > .95 and RMSEA = < .06, when residual covariances between items #1 and #2 and items #5 and #6 were included in the model (see S1 Text for a detailed description of CFA).

#### Measurement invariance

Measurement invariance (MI) between the two datasets (i.e., between 2002 and 2012) and between different subgroups (i.e., defined by questionnaire language and participant gender, age, education level, and domicile) was assessed before any comparisons were made, because scalar measurement invariance is a requirement for the valid contrasting of group means, especially when comparisons are made across time [52].

The MI analysis confirmed that it was feasible to compare mean tightness scores between the ESCP2002 and ESS2012 samples, and also between different subgroups within each sample, because scalar measurement invariance criteria were met, CFIs > .90, RMSEAs < .08 [53]. The only exception was the oldest age group (65–74 years) in the ESCP2002 data and, therefore, this age group is not included in the following across-time and within-country (ESCP2002) comparisons. More information about the measurement invariance analysis can be found in the S1 Text and in S2, S3 and S4 Tables; descriptive statistics about the distribution of respondents among different subgroups are presented in Table 1.

**Table 1:** Mean tightness scores in Estonia in 2002 (ESCP2002) vs. 2012 (ESS2012) in different social subgroups.

	ESCP2002			ESS2012			ESCP2002 *vs*. ESS2012		
Language	*N*	*M*	*SD*	*N*	*M*	*SD*	*F*	*p*	η^2^_p_
Estonian	1328	3.86	0.76	1324	4.02	0.76	28.78	< .001	.008
Russian	254	3.87	0.80	559	4.01	0.70	6.14	.013	.002
**Gender**									
Male	693	3.80	0.79	820	4.02	0.74	31.47	< .001	.009
Female	889	3.91	0.75	1063	4.01	0.75	8.99	.003	.003
**Age**									
15–29	410	3.77	0.65	443	4.00	0.77	20.11	< .001	.008
30–44	430	3.83	0.74	464	4.06	0.68	22.12	< .001	.008
45–59	359	3.89	0.85	526	3.95	0.74	1.48	n.s.	.001
60–74	383	3.98	0.82	450	4.06	0.77	Measurement invariance not achieved		
**Education Level**									
Primary	301	3.95	0.82	215	4.05	0.71	2.39	n.s.	.001
Secondary	713	3.90	0.75	713	4.00	0.78	5.74	.017	.002
Tertiary	568	3.77	0.76	955	4.02	0.72	40.27	< .001	.012
**Place of Residence**									
City	403	3.83	0.78	670	4.00	0.70	13.18	< .001	.004
Town	480	3.85	0.77	623	4.01	0.79	12.71	< .001	.004
Village or farm	690	3.90	0.76	590	4.05	0.74	12.4	< .001	.004

## Results

### Across-time change and within-country variance in tightness scores

**Fig 1:**
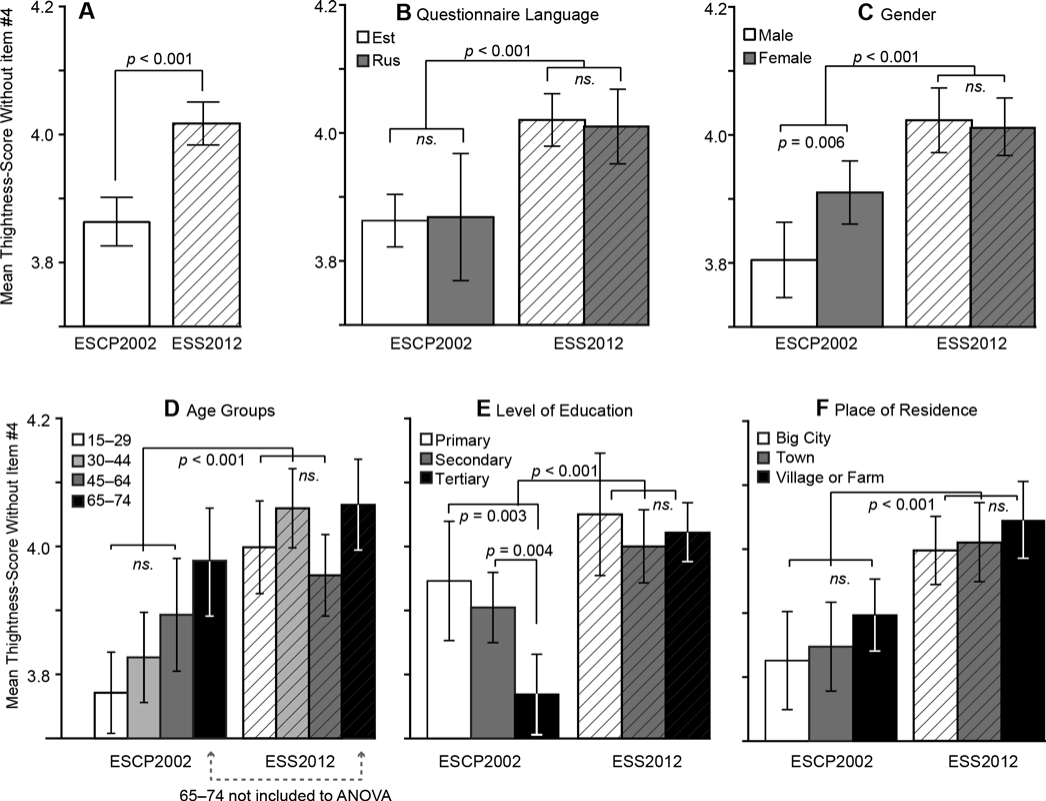
Differences in mean tightness scores in 2002 (ESCP2002) vs. 2012 (ESS2012) as a whole (A) and in different social subgroups: questionnaire language (B), gender (C), age (D), education level (E), and place of residence (F). All *p*-values are Bonferroni-corrected.

Across-time and within-country differences in tightness-looseness scores were tracked with a univariate analysis of variance (ANOVA) and Student’s t-test using SPSS v. 22.0. Fig 1A shows that the mean tightness score is higher in the ESS2012 (*M* = 4.02; *SD* = .74) than in the ESCP2002 data (*M* = 3.86; *SD* = .77). The difference is statistically significant, although the effect size is small (*F*(1, 3463) = 35.49, *p* < .001, η^2^_p_ = .010; all reported *p*-values are Bonferroni-corrected for multiple comparisons). The same tendency is evident across all social subgroups studied (see Table 1). The mean tightness score in the ESCP2002 was the same as the non-standardized tightness score for the Estonian sample (*n* = 188; mean age 32 years; 86% female; 52% students) in a 33-country comparative study [12] for which data were collected in 2001.

To check whether questionnaire language, respondent gender, age, education level, or place of residence would, in addition to sample (ESCP2002 vs. ESS2012), contribute to differences in average tightness scores, these variables were added one-by-one into a two-way factorial ANOVA model (together with the *sample* factor).

As there were no significant differences in average tightness scores between people who had filled in the questionnaire in Estonian and those who had filled it in in Russian (Fig 1B; see Table 1 *Language* for descriptive statistics), respondents are not separated by language in further analysis.

As expected, the main effect of *sample* (ESCP2002 vs. ESS2012) was significant in all comparisons (*F*(1, 2626–3461) = 21.65–38.34, *p* < .001, η^2^_p_ = .006–.011). In addition to *sample*, only *education level* (see Fig 1E) showed a significant main effect (*F*(2, 3459) = 4.08, *p* = .017, η^2^_p_ = .002), with the average tightness score being higher for people who have a lower education level, but the difference was significant only between primary and tertiary education (for all mean values, see Table 1).

Several other factors showed statistically significant interactions with *sample*. The interaction between *sample* and *gender* (*F*(1, 3461) = 4.96, *p* = .026, η^2^_p_ = .001) indicated that tightness in men had risen more than in female respondents (all mean values are presented in Table 1) over the period of ten years, and the difference between men and women was significant only in the ESCP2002 data (*F*(1, 1580) = 7.60, *p* = .006, η^2^_p_ = .002).

The interaction between *sample* and *respondent age* (*F*(2, 2626) = 3.72, *p* = .024, η^2^_p_ = .003) revealed that tightness scores in the ten years had risen the most among 15–29 (*F*(1, 2626) = 20.1 *p* < .001, η^2^_p_ = .008) and 30–44 year-old respondents (*F*(1, 2626) = 22.1 *p* < .001, η^2^_p_ = .008). There were no significant differences in tightness scores between the age groups either in the ESCP2002 or in the ESS2012 sample. As already mentioned, the group of 60–74-years-olds was not included in the ANOVA analysis, because the scalar measurement invariance assumption was not fulfilled for this group.

*Sample* and *level of education* also revealed a significant interaction in average tightness scores (*F*(2, 3459) = 4.37, *p* = .013, η^2^_p_ = .003), meaning that, in 2002, tightness scores decreased as education level increased, but, in the ESS2012 sample, there was no significant difference in tightness between the three education levels. Overall tightness rose significantly among people with secondary (*F*(1, 3459) = 5.74, *p* = .017, η^2^_p_ = .002) and tertiary education (*F*(1, 3459) = 40.27, *p* < .001, η^2^_p_ = .012).

#### Across-time change in single-item responses

**Fig 2:**
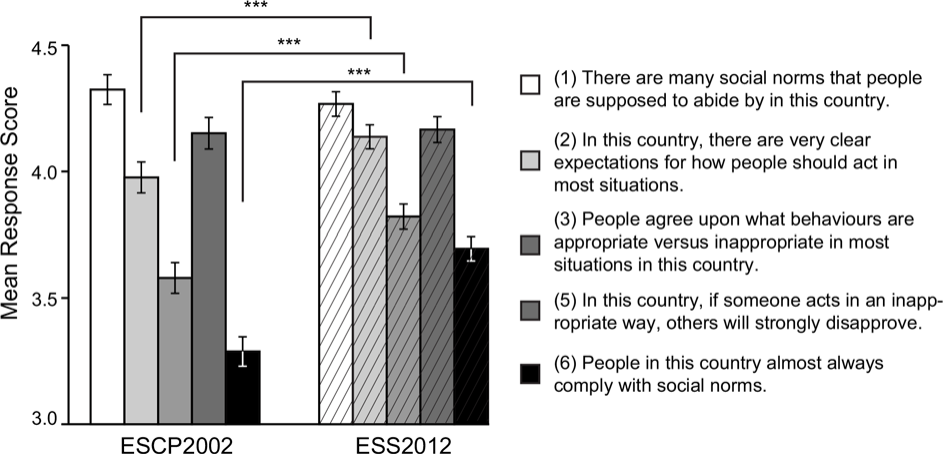
Average response scores to five TLS items (item #4 omitted) in 2002 (ESCP2002) vs. 2012 (ESS2012). ^***^ *p* < .001.

Finally, in order to better understand the factors behind the rise in the mean tightness score in Estonia from 2002 to 2012, changes in response to single TLS items were tracked. Fig 2 shows the mean (± 95% confidence intervals indicated by error bars) responses to single TLS items, separately for the ESCP2002 (left) and ESS2012 (right) samples. It is evident that in 2012 people still agreed that there are rather many social norms in Estonia (item #1) and that people will disapprove of inappropriate behavior (item #5). These perceptions had not changed in the ten years.

At the same time, in the year 2012, people reported having clearer expectations of how they should act in certain situations (item #2; ESCP2002 *M* = 3.98. *SD* = 1.25, ESS2012 *M* = 4.14, *SD* = 1.06; *t*(3115) = -4.06, *p* < .001, *d* = -0.14) and stronger general agreement about appropriate and inappropriate behaviors (item #3; ESCP2002 *M* = 3.58. *SD* = 1.24, ESS2012 *M* = 3.82, *SD* = 1.11; *t*(3202) = -6.01, *p* < .001, *d* = -0.20) than in 2002. Also, compliance with social norms had significantly risen (item #6; ESCP2002 *M* = 3.29. *SD* = 1.18, ESS2012 *M* = 3.70, *SD* = 1.07; *t*(3219) = -10.64, *p* < .001, *d* = -0.34).

## Discussion

Tightness-looseness, defined as the strength of social norms and tolerance of deviant behavior, was recently proposed as a core or critical dimension along which cultures vary [12]. Cultural tightness–looseness has even been shown to moderate the effects of cultural values at the national level, among many other things, with Hofstede’s individualism–collectivism and power distance having significantly stronger effects on various outcomes in culturally tighter countries [54]. However, as cultures are not static but “constantly changing, open systems of attitudes, norms, behaviors, artefacts, and institutions that people reinforce but also continually modify or even challenge through diverse means of participation and engagement” (p. 789, [55]), it is also important to examine change across time as well as within-country variation in tightness-looseness in order to understand and model cultural change. The current study was undertaken to fill this substantial gap in our knowledge. Specifically, we examined across-time variation and within-country differences in tightness-looseness by surveying two large and nationally representative samples of Estonians about their perception of tightness-looseness in their country over a period of 10 years, that is, from 2002 to 2012.

### Across-time change in tightness-looseness

Measurement invariance analyses showed that cultural tightness-looseness is a valid and reliable cultural construct, with a stable structure over a ten-year period, across and within different social subgroups. The mean tightness score in 2002 (*M* = 3.86) was the same as the non-standardized tightness score for the Estonian convenience sample in a 33-country comparative study [12] for which data were collected in 2001. Thus, our findings show that, even in loose countries, such as Estonia, where norms, values, and behaviors show greater variability than in tight countries, convenience or unrepresentative samples may adequately reflect the general population (see [21], for a differing view).

In terms of across-time change in tightness-looseness, the results of the current study show that cultural tightness in Estonia rose significantly from 2002 to 2012, although the effect size of the change was small. It is argued by several theorists as well as demonstrated by empirical research (e.g., [22, 25, 56]) that socioeconomic development in a society brings systematic and pervasive cultural changes, including changes in people’s basic value orientations. In 2002, when our first data were collected, Estonia was still a transitional country, on a path of re-integration with Europe, with joining the EU and NATO in 2004 marking the end of the transition period [36]. The economy grew rapidly until 2007, then declined considerably during the financial crisis of 2008–2009, and began to slowly rise again during 2010–2012. We cannot say with any certainty whether, or to what extent, the slight rise in tightness that we saw in 2012, as compared to 2002, is due to changes in social and/or economic development in Estonian society during this period. It is, however, highly likely that tightness scores rose from 2002 to 2012 because new rules and norms for behavior (the previous ones were turned upside-down when Estonia regained its independence in 1991) had finally become established. This interpretation is further supported by the item-level analysis of the TLS, which revealed that, whereas the perceived number of social norms had not changed from 2002 to 2012, people felt that the norms were clearer and there was more general agreement about appropriate vs. inappropriate behavior in 2012 than in 2002. The expected compliance with social norms had also risen significantly. Thus, this perceived clarification of social norms can, on the one hand, be viewed as an indication of cultural stabilization (e.g., related to the end of the transition phase in Estonia), but, on the other hand, can explain the rise in cultural tightness–there is now more agreement about what appropriate behaviors in this culture are.

To sum up, the current study showed that the level of perceived tightness in a culture can change over a period of ten years. However, despite the fact that Estonian society has undergone some major changes during the time period studied, the effect size of the change in the mean tightness score was relatively small. Thus, our results suggest that tightness-looseness, similarly to cultural value orientations, is a relatively stable and robust characteristic of culture–change indeed takes place, but slowly [42, 46, 57]. However, further studies need to be conducted to test these findings in other countries with either a more stable or a more turbulent economic and political history, in order to find out whether, and how much, changes in social structure, politics, or economic growth/decline affect the perceived strength of social norms in a culture.

### Within country-differences in tightness-looseness

Although the mean tightness score in Estonia had risen slightly from 2002 to 2012, the small within-country differences that were present in 2002 (tightness scores were slightly higher for females than males, and lower for people who had higher education compared with those who had primary or secondary education) had disappeared by 2012. The fact that social norms were perceived more clearly and there was more agreement about appropriate vs. inappropriate behavior in 2012 than 2002 might also explain why the within-country differences that were present in 2002 had vanished by 2012. Yet, the main finding of our study is that despite Estonia being a loose country, which, by definition, means that is has greater within-country heterogeneity in norms, behaviors, and values than tight countries, we were not able to identify any significant within-country differences in TLS in terms of age, gender, place of residence, education, or ethnicity. But, as already noted, Estonia is a relatively loose, yet a very small country, and therefore, further research about intracultural variation in tightness-looseness should target larger societies with more diverse populations. For example, a recent study [16], using a theoretically constructed index of tightness-looseness, showed that within-country differences in large countries, where people come from varying cultural backgrounds, can be evident.

Clearly, the most surprising finding of this study was that we did not find any significant differences in tightness-looseness between the Estonian-speaking majority (roughly 68%) and Russian-speaking minority populations. This finding is especially remarkable as many previous studies have shown that there are large differences in value preferences between the Estonian- and Russian-speaking populations in Estonia [38, 58, 59]. Also, one might have expected that the so-called “Bronze soldier crisis” that took place in 2007 [60, 61] would have shifted perceptions of tightness-looseness among the Estonian- and Russian-speaking populations in different directions. However, similarly to 2002, there were no differences in perceived social norms between ethnic Estonians and Estonian Russians in 2012, and the overall agreement about acceptable and unacceptable behavior had risen. Thus, although a recent report [62] shows that there is still a strong division across linguistic, ethnic, and socioeconomic lines in Estonia, with Estonian- and Russian-speakers living in different ‘cultural spaces’ (both in terms of residential and work-place segregation, as well in media use), the two ethnolinguistic groups seem to share the same perception of the strength of social norms and deviant behavior in their country. However, one should bear in mind here that the measure of tightness-looseness that we used in our study, the TLS, only tells us about the perceived strength and clarity of social norms in Estonia but not about the content or specific expressions of these social norms. Thus, while Estonian- and Russian-speakers agree about the level of societal permissiveness in Estonia, their values and social norms might still be different.

In conclusion, our results suggest that tightness-looseness is a relatively stable and robust characteristic of culture with low levels of within-country variation, at least in small and culturally uniform settings, such as Estonia. Future studies about across-time change and within-country variance in tightness-looseness should target more culturally diverse and socially divided societies.

## Supporting Information

S1 Appendix Items of the Tightness-Looseness Scale. (PDF)

S1 Text Reliability and validity of the Tightness-Looseness Scale. (PDF)

S1 Table Inter-item correlations between the items of the Tightness-Looseness Scale. (PDF)

S2 Table Results of measurement invariance tests of the Tightness-Looseness Scale between ESCP2002 and ESS2012 data. (PDF)

S3 Table Results of measurement invariance tests of the Tightness-Looseness Scale between different subgroups in the ESCP2002 data. (PDF)

S4 Table Results of measurement invariance tests of the Tightness-Looseness Scale between different subgroups in the ESS2012 data. (PDF)
