# Supplementary material for: Across-Time Change and Variation in Cultural Tightness-Looseness
Source: PLoS One. 2015 Dec 18;10(12):e0145213. doi: 10.1371/journal.pone.0145213 (PMC4684201; doi:10.1371/journal.pone.0145213)
Supplement: S1 Appendix — (PDF) [file pone.0145213.s001.pdf]

## S1 Appendix: Items of the Tightness-Looseness Scale

## Article: "Across-Time Change and Variation in Cultural Tightness-Looseness"

**Authors:** Anne Mandel[1] and Anu Realo[2*]
[1]Brain Research Unit, O.V. Lounasmaa Laboratory, Aalto University, Helsinki, Finland
[2]Department of Psychology, University of Tartu, Tartu, Estonia
[*]anu.realo@ut.ee

## The original English items of the Tightness-Looseness Scale (Gelfand et al., 2011[1])

The following statements refer to a country as a whole. Please indicate whether you agree or disagree with the following statements using the following scale. Note that the statements sometimes refer to "social norms," which are standards for behavior that are generally unwritten.

| **1** *Strongly disagree* | **2** *Moderately disagree* | **3** *Slightly disagree* | **4** *Slightly agree* | **5** *Moderately agree* | **6** *Strongly agree* |
|---|---|---|---|---|---|

1. There are many social norms that people are supposed to abide by in this country.
2. In this country, there are very clear expectations for how people should act in most situations.
3. People agree upon what behaviors are appropriate versus inappropriate in most situations this country.
4. People in this country have a great deal of freedom in deciding how they want to behave in most situations.
5. In this country, if someone acts in an inappropriate way, others will strongly disapprove.
6. People in this country almost always comply with social norms.

[1] Gelfand, M. J., Raver, J. L., Nishii, L., Leslie, L. M., Lun, J., Lim, B. C., . . . Yamaguchi, S. (2011). Differences between tight and loose cultures: A 33-nation study. *Science, 332*(6033), 1100-1104. doi:10.1126/science.1197754

## The Estonian version of the Tightness-Looseness Scale

**Järgnevad laused käivad Eesti kohta tervikuna. Teie ülesandeks on hinnata, kas nõustute järgnevate lausetega või mitte. Pange tähele, et mõned laused viitavad “sotsiaalsetele normidele” – nende all mõeldakse niiöelda kirjutamata reegleid käitumisstandardite kohta, mis ühes või teises olukorras võivad kehtida. Vastamiseks tõmmake palun ring ümber ühele, Teie jaoks sobilikuima vastusevariandi numbrile iga väite järel, kasutades järgmist skaalat:**

| **1**<br>*ei ole üldse nõus* | **2**<br>*enamalt jaolt ei ole nõus* | **3**<br>*pigem ei ole nõus* | **4**<br>*pigem nõus* | **5**<br>*enamalt jaolt nõus* | **6**<br>*täiesti nõus* |
|---|---|---|---|---|---|

| | | | | | | | |
|---|---|---|---|---|---|---|---|
| 1 | Eestis on palju sotsiaalseid norme, millest inimesed peaksid kinni pidama | 1 | 2 | 3 | 4 | 5 | 6 |
| 2 | Eestis on enamikes situatsioonides käitumise kohta selged reeglid | 1 | 2 | 3 | 4 | 5 | 6 |
| 3 | Eestis elavad inimesed on ühel nõul selles suhtes, millised on enamikes situatsioonides sobilikud käitumisviisid ja millised mitte | 1 | 2 | 3 | 4 | 5 | 6 |
| 4 | Eestis elavatel inimestel on suur vabadus otsustada, kuidas nad enamikes situatsioonides käituda tahavad | 1 | 2 | 3 | 4 | 5 | 6 |
| 5 | Kui keegi käitub ebasobivalt, mõistavad teised selle kindlasti hukka | 1 | 2 | 3 | 4 | 5 | 6 |
| 6 | Eestis elavad inimesed käituvad peaaegu alati vastavalt sotsiaalsetele normidele | 1 | 2 | 3 | 4 | 5 | 6 |

## The Russian version of the Tightness-Looseness Scale

**Нижеследущие предложения касаются Эстонии в целом. Ваша задача состоит в том, чтобы оценить, согласны ли Вы с данными предложениями. Пожалуйста заметьте, что некоторые вопросы намекают на “социальные нормы” – под этим подразумеваются так называемые неписанные правила стандартов поведения, которые могут быть действительны в той или другой ситуации. Отвечая обведите пожалуйста номер одного, наиболее подходящего для Вас варианта ответа за каждым утверждением, используя данную шкалу:**

| **1**<br>*совершенно не согласен* | **2**<br>*в основном не согласен* | **3**<br>*скорее не согласен* | **4**<br>*скорее согласен* | **5**<br>*в основном согласен* | **6**<br>*абсолютно согласен* |
|---|---|---|---|---|---|

| | | | | | | | |
|---|---|---|---|---|---|---|---|
| 1 | В Эстонии много социальных норм, которых люди должны бы придерживатся | 1 | 2 | 3 | 4 | 5 | 6 |
| 2 | В Эстонии в большинстве случаев есть конкретные правила поведения | 1 | 2 | 3 | 4 | 5 | 6 |
| 3 | Люди, живущие в Эстонии единогласны в мнении о том, какое поведение подходит и какое не подходит для большинства случаев | 1 | 2 | 3 | 4 | 5 | 6 |
| 4 | У людей, живущих в Эстонии, есть большая свобода решать, как они желают вести себя в большинстве ситуаций | 1 | 2 | 3 | 4 | 5 | 6 |
| 5 | Если кто-либо ведет себя неподобающим образом, другие обязательно отнесутся к этому с осуждением | 1 | 2 | 3 | 4 | 5 | 6 |
| 6 | Люди, живущие в Эстонии ведут себя почти всегда соответственно социальным нормам | 1 | 2 | 3 | 4 | 5 | 6 |
