# Supplementary material for: Across-Time Change and Variation in Cultural Tightness-Looseness
Source: PLoS One. 2015 Dec 18;10(12):e0145213. doi: 10.1371/journal.pone.0145213 (PMC4684201; doi:10.1371/journal.pone.0145213)
Supplement: S1 Text — (PDF) [file pone.0145213.s002.pdf]

S1 Text: Reliability and validity of the Tightness-Looseness Scale

Article: "Across-Time Change and Variation in Cultural Tightness-Looseness"

Authors: Anne Mandel[1] and Anu Realo[2*]
[1]Brain Research Unit, O.V. Lounasmaa Laboratory, Aalto University, Helsinki, Finland
[2]Department of Psychology, University of Tartu, Tartu, Estonia
[*]anu.realo@ut.ee

Model Fit

A confirmatory factor analysis (CFA) was used to test whether the single-factor tightness-looseness model [1] fit the current data. In the ESCP2002 data, factor loadings of the items ranged from .33 (item 5) to .69 (item 2) in the initial 5-item model, and the model did not reach an acceptable fit in an adjusted goodness of fit index, AGFI = .92, CFI = .89, and RMSEA = .11, when the following criteria for evaluation of the model fit were used: comparative fit index (CFI) and adjusted comparative fit index (AGFI) > .90 and root means square error of approximation (RMSEA) < .08 [2]. Modification indices showed strong covariances between the residuals of items #1 and #2 (modification index (mod. ind.) = 44.8 and parameter change (par. ch.) = 0.2), and between the residuals of items #5 and #6 (mod. ind. = 30.6 and par. ch. = 0.2).

After adding the covariance between items #1 and #2, the model fit was very good (AGFI = .98, CFI = .98, RMSEA = .06) and the factor loadings of the items ranged from .26 (item #1) to .68 (item #3). The covariance value between items #1 and #2 was .29. When covariances between items #1 and #2 and items #5 and #6 were included, the modified model showed a nearly perfect fit to the data (AGFI = .997,CFI = 1.00, RMSEA < .00); the factor loadings in the modified model ranged from .26 (item #1) to .72 (item #3), with the residual covariance values being .29 between items #1 and #2 and .13 between items #5 and #6.

In the ESS2012 data, the factor loadings of the 5 items ranged from .40 (item #6) to .80 (item #2) in the initial model, but, as in the ESCP2002 data, the model failed to reach an acceptable level of fit (AGFI = .87, CFI = .89, RMSEA = .14). Modification indices again highlighted error covariances between several items, the strongest covariance being between items #5 and #6 (mod. ind. = 89.4, par. ch. = 0.23). Allowing residual covariance between items #5 and #6 did not lead to a clearly acceptable model fit (AGFI = .92, CFI = .94, RMSEA = .12). Using the same modification that was used for the ESCP2002 data and adding covariances between items #1 and #2 and items #5 and #6 to the model improved the fit considerably (AGFI = .97, CFI = .99 and RMSEA = .06). The item factor loadings in the modified model varied between .43 (item 5) and .71 (item 3). The residual covariance value between items 1 and 2 was .40 and between items 5 and 6 was .18.

Testing Across-time and Within-Country Measurement Invariance (MI)

SPSS Amos (v. 22) was used to establish measurement invariance (MI) over time (i.e., between 2002 and 2012) and across different social subgroups by the following: a) questionnaire language (*Estonian*, *Russian*); b) gender (*male*, *female*); c) age (*15-29*, *30-44*, *45-59*, *60-74*; groups optimized to balance the number of respondents in each group and to roughly correspond to different life stages: studying and specializing, early and late working life, retirement); d) education level (*primary*: 1-9 years of education; *secondary*: 10-12 years of education; and *tertiary*: 13 or more years of education); and e) domicile (*city and suburbs*, *town*, *country village or farm*).

The following single-factor MI model was constructed [see 2], where *response to a questionnaire item* (*X*) would be defined as

$$X = b_0 + b_1 \text{ x ksi} + b_2 \text{ x error (Equation 1).}$$

In Equation 1 (Eq. 1), b_0 indicates the item intercept, b_1 the regression coefficient (factor loading in the standard solution), *ksi* the latent variable (tightness-looseness), and b_2 the regression coefficient of the residual variance (*error*).

To compare factor loadings across groups, the latent factor (*ksi*) mean was constrained to 0 and its variance made equal to 1. Also, the regression coefficients of the residual variance were fixed to 1 (b_2 in Eq. 1), and the means of the residual variances were fixed to 0.

MI was measured at four different levels: 1) *configural MI*, which confirms that the proposed single-factor model is valid in each of the subgroups studied in both of the datasets; 2) *metric MI*, which adds the assumption that the factor loadings of the items are equal across groups (b_1 in Eq. 1), 3); and *scalar MI*, which adds the assumption that item intercepts (b_0 in Eq. 1) are also equal across groups. Scalar MI allows the conducting of comparisons of means between the datasets and across the different subgroups [3, 4]. In addition, *strict MI* (sometimes called *full uniqueness MI*) means that residual variances (error in Eq. 1) are also equal across groups.

First, in order to find out if it is possible to compare the mean tightness scores from the ESCP2002 with those from the ESS2012, the MI of the TLS was tested to establish the extent to which the scale produces comparable results over time [see 3]. Based on the above-described CFA models, we used the single-factor model (with 5 items) for the TLS and allowed the residuals of items #1 and #2 and items #5 and #6 to covary.

A comparison between ESCP2002 and ESS2012 datasets indicated an acceptable fit at the scalar MI level (CFI = .93, RMSEA = .06), which, as already mentioned, is a necessary precondition for comparing mean scores. In the ESCP2002 vs. ESS2012 subgroup-comparisons (e.g., males in ESCP2002 vs. ESS2012), scalar MI criteria (CFI > .9, RMSEA < .08)

were met for all subgroups, except the oldest age group (65–74 years). S2 Table gives an overview of the detailed results.

One of the aims of the current study was to probe within-country variance in tightness scores. Therefore, MI tests within surveys were necessary to find out whether comparisons of mean tightness scores of different subgroups were feasible. The subgroups used for categorization, as indicated above, were *Questionnaire Language, Gender, Age, Education Level, and Place of Residence*. In the ESCP2002 sample, all subgroups met strict MI criteria (all CFI-s > .90, all RMSEA-s < .06; see S3 Table for detailed results), except for the oldest subgroup (65–74 years) of the *Age* variable, which had to be left out to meet acceptable criteria (both at the scalar and strict MI levels). In the ESS2012 data, all subgroups met the strict MI criteria (all CFI-s > .97, all RMSEA-s ≤ .05; see S4 Table for detailed results).

To sum up, the MI analysis showed that it is feasible to compare mean tightness scores between the ESCP2002 and ESS2012 samples, and also between different subgroups within both samples, because scalar MI criteria were met. The only exception was the oldest age group (65–74 years) in the ESCP2002 data and, therefore, this age group is not included in the across-time and within-country (ESCP2002) comparisons.

References

1. Gelfand MJ, Raver JL, Nishii L, Leslie LM, Lun J, Lim BC, et al. Differences between tight and loose cultures: A 33-nation study. *Science*. 2011;332(6033):1100-4. doi: 10.1126/science.1197754. PubMed PMID: WOS:000290996700049.
2. Van de Schoot R, Lugtig P, Hox J. A checklist for testing measurement invariance. *European Journal of Developmental Psychology*. 2012;9:486-92.
3. Davidov E. A cross-country and cross-time comparison of the human values measurements with the second round of the European Social Survey. *Survey Research Methods*. 2008;2:33-46.
4. Meredith W. Measurement invariance, factor analysis and factorial invariance. *Psychometrika*. 1993;58:525-43.
