# Supplementary material for: Across-Time Change and Variation in Cultural Tightness-Looseness
Source: PLoS One. 2015 Dec 18;10(12):e0145213. doi: 10.1371/journal.pone.0145213 (PMC4684201; doi:10.1371/journal.pone.0145213)
Supplement: S1 Table — (PDF) [file pone.0145213.s003.pdf]

S1 Table

Inter-item correlations between the items of the Tightness-Looseness Scale

ESCP2002	Item 2	Item 3	Item 4	Item 5	Item 6
Item 1	.38**	.19**	.12**	.12**	.09**
Item 2		.41**	.07*	.19**	.26**
Item 3			.13**	.22**	.32**
Item 4				.00	.16**
Item 5					.26**
ESS2012					
Item 1	.58**	.33**	.24**	.26**	.18**
Item 2		.45**	.22**	.29**	.28**
Item 3			.24**	.28**	.35**
Item 4				.22**	.18**
Item 5					.34**

* p = .003; ** p < .0001
