# Supplementary material for: Across-Time Change and Variation in Cultural Tightness-Looseness
Source: PLoS One. 2015 Dec 18;10(12):e0145213. doi: 10.1371/journal.pone.0145213 (PMC4684201; doi:10.1371/journal.pone.0145213)
Supplement: S2 Table — (PDF) [file pone.0145213.s004.pdf]

S2 Table

Measurement invariance of the Tightness-Looseness Scale between ESCP2002 and ESS2012 data: Single-factor model with 5 items (item #4 excluded; items #1 and #2, and items #5 and #6 covarying)

ESCP2002* vs. *ESS2012	χ^2	*df*	*p*	CFI	RMSEA	AIC
Configural	26.97	6	.000	.99	.03	94.97
Metric	58.15	11	.000	.98	.04	116.15
Scalar	207.25	16	.000	.93	.06	255.25
Strict	370.33	21	.000	.87	.07	408.34

Comparisons between *ESCP2002* vs. *ESS2012* samples by different subgroups

**Questionnaire language**						
Estonian	χ^2	*df*	*p*	CFI	RMSEA	AIC
Configural	32.93	6	.000	.99	.04	100.93
Metric	76.12	11	.000	.97	.05	134.12
Scalar	227.94	16	.000	.91	.07	275.94
Strict	336.16	21	.000	.86	.08	374.16
Russian	χ^2	*df*	*p*	CFI	RMSEA	AIC
Configural	8.84	6	.183	1.00	.02	76.84
Metric	22.81	11	.019	.98	.04	80.81
Scalar	64.12	16	.000	.92	.06	112.13
Strict	129.69	21	.000	.81	.08	167.69

**Gender**						
Male	χ^2	*df*	*p*	CFI	RMSEA	AIC
Configural	19.52	6	.003	.99	.04	87.52
Metric	22.88	11	.018	.99	.03	80.88
Scalar	105.23	16	.000	.93	.06	153.23
Strict	195.62	21	.000	.85	.07	233.62
Female						
Configural	16.85	6	.010	.99	.03	84.85
Metric	59.18	11	.000	.97	.05	117.18
Scalar	130.19	16	.000	.93	.06	178.19
Strict	213.65	21	.000	.88	.07	251.65

S2 Table (continues)

Age						
15-29	χ^2	*df*	*p*	**CFI**	**RMSEA**	**AIC**
Configural	13.16	6	.041	.99	.04	81.16
Metric	32.34	11	.001	.97	.05	90.34
Scalar	68.12	16	.000	.92	.06	116.12
Strict	103.66	21	.000	.88	.07	141.66
30-44						
Configural	6.56	6	.364	1.00	.01	74.56
Metric	20.13	11	.044	.99	.03	78.13
Scalar	78.78	16	.000	.90	.07	126.78
Strict	111.17	21	.000	.86	.07	149.17
45-59						
Configural	9.59	6	.143	1.00	.03	77.59
Metric	20.59	11	.038	.99	.03	78.59
Scalar	49.80	16	.000	.95	.05	97.81
Strict	106.20	21	.000	.88	.07	144.21
60-74						
Configural	19.94	6	.003	.98	.05	87.94
Metric	57.18	11	.000	.94	.07	115.17
Scalar	112.37	16	.000	.87	.09	160.37
Strict	157.38	21	.000	.82	.09	195.38

**Level of Education**						
Primary	χ^2	*df*	*p*	**CFI**	**RMSEA**	**AIC**
Configural	18.93	6	.004	.96	.07	86.93
Metric	24.53	11	.011	.96	.05	82.53
Scalar	33.24	16	.007	.95	.05	81.25
Strict	73.63	21	.000	.84	.07	111.63
Secondary	χ^2	*df*	*p*	**CFI**	**RMSEA**	**AIC**
Configural	12.15	6	.059	.99	.03	80.15
Metric	35.87	11	.000	.98	.04	93.87
Scalar	100.86	16	.000	.92	.06	148.86
Strict	137.90	21	.000	.89	.06	175.90
Tertiary						
Configural	10.34	6	.111	1.00	.02	78.34
Metric	27.87	11	.003	.99	.03	85.87
Scalar	143.23	16	.000	.91	.07	191.23
Strict	199.56	21	.000	.87	.08	237.56

S2 Table (continues)

Place of residence						
City & suburbs	χ^2	*df*	*p*	**CFI**	**RMSEA**	**AIC**
Configural	13.83	6	.032	.99	.04	81.83
Metric	21.73	11	.027	.99	.03	79.73
Scalar	56.28	16	.000	.95	.05	104.28
Strict	127.84	21	.000	.87	.07	165.84
Town						
Configural	12.61	6	.050	.99	.03	80.62
Metric	25.48	11	.008	.99	.04	83.49
Scalar	80.12	16	.000	.94	.06	128.12
Strict	117.54	21	.000	.90	.07	155.54
Village or farm						
Configural	12.58	6	.050	.99	.03	80.58
Metric	3.82	11	.001	.98	.04	88.82
Scalar	90.70	16	.000	.92	.06	138.70
Strict	159.76	21	.000	.85	.07	197.76
