# Supplementary material for: Across-Time Change and Variation in Cultural Tightness-Looseness
Source: PLoS One. 2015 Dec 18;10(12):e0145213. doi: 10.1371/journal.pone.0145213 (PMC4684201; doi:10.1371/journal.pone.0145213)
Supplement: S3 Table — (PDF) [file pone.0145213.s005.pdf]

S3 Table

Measurement invariance of the Tightness-Looseness Scale between different subgroups in the ESCP2002 data: Single-factor model with 5 items (item #4 excluded; items #1 and# 2, and items #5 and #6 covarying)

	χ^2	*df*	*p*	CFI	RMSEA	AIC
**Questionnaire language**						
Configural	7.71	6	.260	1.00	.01	75.71
Metric	21.34	11	.030	.99	.02	79.34
Scalar	91.89	16	.000	.92	.06	139.89
Strict	108.96	21	.000	.91	.05	146.96
**Gender**						
Configural	6.09	6	.413	1.00	.00	74.09
Metric	11.62	11	.393	1.00	.01	69.62
Scalar	19.78	16	.230	1.00	.01	67.78
Strict	30.10	21	.090	.99	.02	68.10
**Age**						
Age group 65–74						
Configural	19.31	12	.081	.99	.02	155.31
Metric	78.20	27	.000	.94	.04	184.20
Scalar	149.83	42	.000	.88	.04	225.83
Strict	203.57	63	.000	.85	.04	237.57
Age group 65–74 excluded						
Configural	9.08	9	.430	1.00	.00	111.08
Metric	35.91	19	.011	.97	.03	117.91
Scalar	55.29	29	.002	.96	.03	117.29
Strict	101.76	43	.000	.91	.03	135.76
**Level of Education**						
Configural	9.57	9	.386	1.00	.01	111.57
Metric	18.92	19	.462	1.00	.00	100.92
Scalar	70.94	29	.000	.95	.03	132.94
Strict	119.98	39	.000	.91	.04	161.98
**Place of Residence**						
Configural	7.46	9	.589	1.00	.00	109.46
Metric	13.92	19	.789	1.00	.00	95.92
Scalar	29.31	29	.449	1.00	.00	91.31
Strict	34.09	39	.693	1.00	.00	76.09
