# Supplementary material for: Across-Time Change and Variation in Cultural Tightness-Looseness
Source: PLoS One. 2015 Dec 18;10(12):e0145213. doi: 10.1371/journal.pone.0145213 (PMC4684201; doi:10.1371/journal.pone.0145213)
Supplement: S4 Table — (PDF) [file pone.0145213.s006.pdf]

S4 Table

Measurement invariance of the Tightness-Looseness Scale between different subgroups in the ESS2012 data: Single-factor model with 5 items (item #4 excluded; items #1 and# 2, and items #5 and #6 covarying)

**Questionnaire language**	χ^2	*df*	*p*	**CFI**	**RMSEA**	**AIC**
Configural	34.05	6	.000	.99	.05	102.05
Metric	55.02	11	.000	.98	.05	113.02
Scalar	60.47	16	.000	.98	.04	108.85
Strict	63.56	21	.000	.98	.03	101.56
**Gender**						
Configural	30.29	6	.000	.99	.05	98.29
Metric	41.69	11	.000	.98	.04	99.69
Scalar	45.17	16	.000	.98	.03	93.17
Strict	55.26	21	.000	.98	.03	93.26
**Age**						
Configural	29.94	12	.003	.99	.03	165.94
Metric	50.92	27	.004	.99	.02	156.92
Scalar	73.37	42	.002	.98	.02	149.37
Strict	112.03	63	.000	.97	.16	146.03
**Level of Education**						
Configural	31.88	9	.000	.99	.04	133.84
Metric	44.37	19	.001	.99	.03	126.37
Scalar	52.51	29	.005	.99	.02	114.51
Strict	85.32	39	.000	.98	.03	127.32
**Place of Residence**						
Configural	31.57	9	.000	.99	.04	133.57
Metric	47.15	19	.000	.99	.03	129.15
Scalar	53.26	29	.004	.99	.02	115.26
Strict	71.91	39	.001	.98	.02	113.91
